# Supplementary figures and images for: Ovalbumin-specific regulatory T cells differentiated from the naïve phenotype (CD44loCD62Lhi) in mesenteric lymph nodes stably suppress enteropathy even in severe food-allergic mice
Source: PLoS One. 2025 May 30;20(5):e0324105. doi: 10.1371/journal.pone.0324105 (PMC12124522; doi:10.1371/journal.pone.0324105)

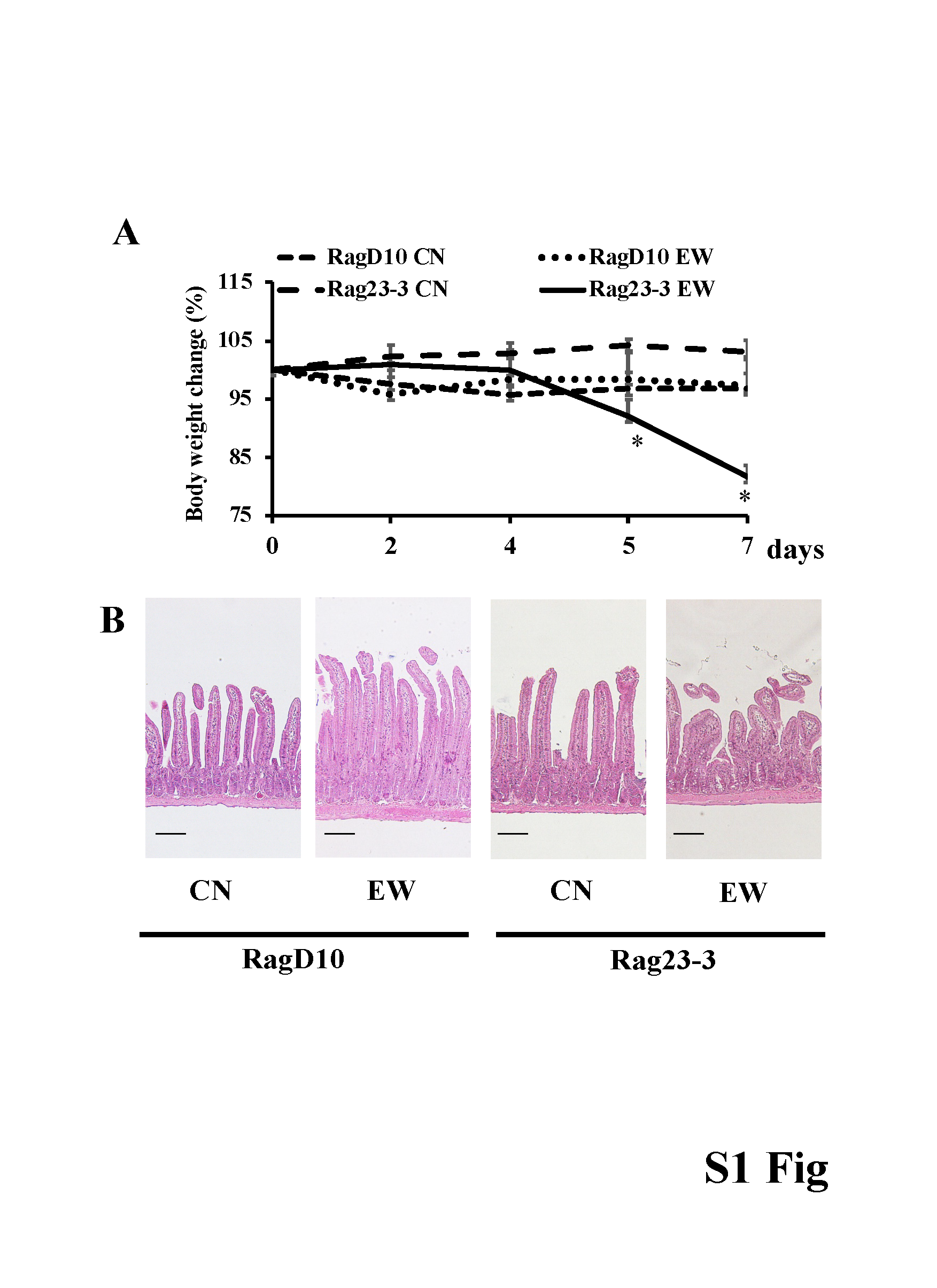

Supplement: S1 Fig — A) RagD10 and Rag23−3 mice were fed a diet containing EW or CN for 7 days, and body weights were measured on days 0, 2, 4, 5, and 7. Body weight changes were determined relative to those on day 0. Values are expressed as mean ± SD. B) Jejunum histology. Samples of jejunum were collected from the mice on day 7 and stained with hematoxylin and eosin. Scale bars, 100 µm. n = 3 per group. Data are representative of two independent experiments. Statistical analysis: Tukey’s HSD test [*p < 0.05 (Rag23−3 EW vs each of the three groups)]. (TIFF) [file pone.0324105.s001.tiff]

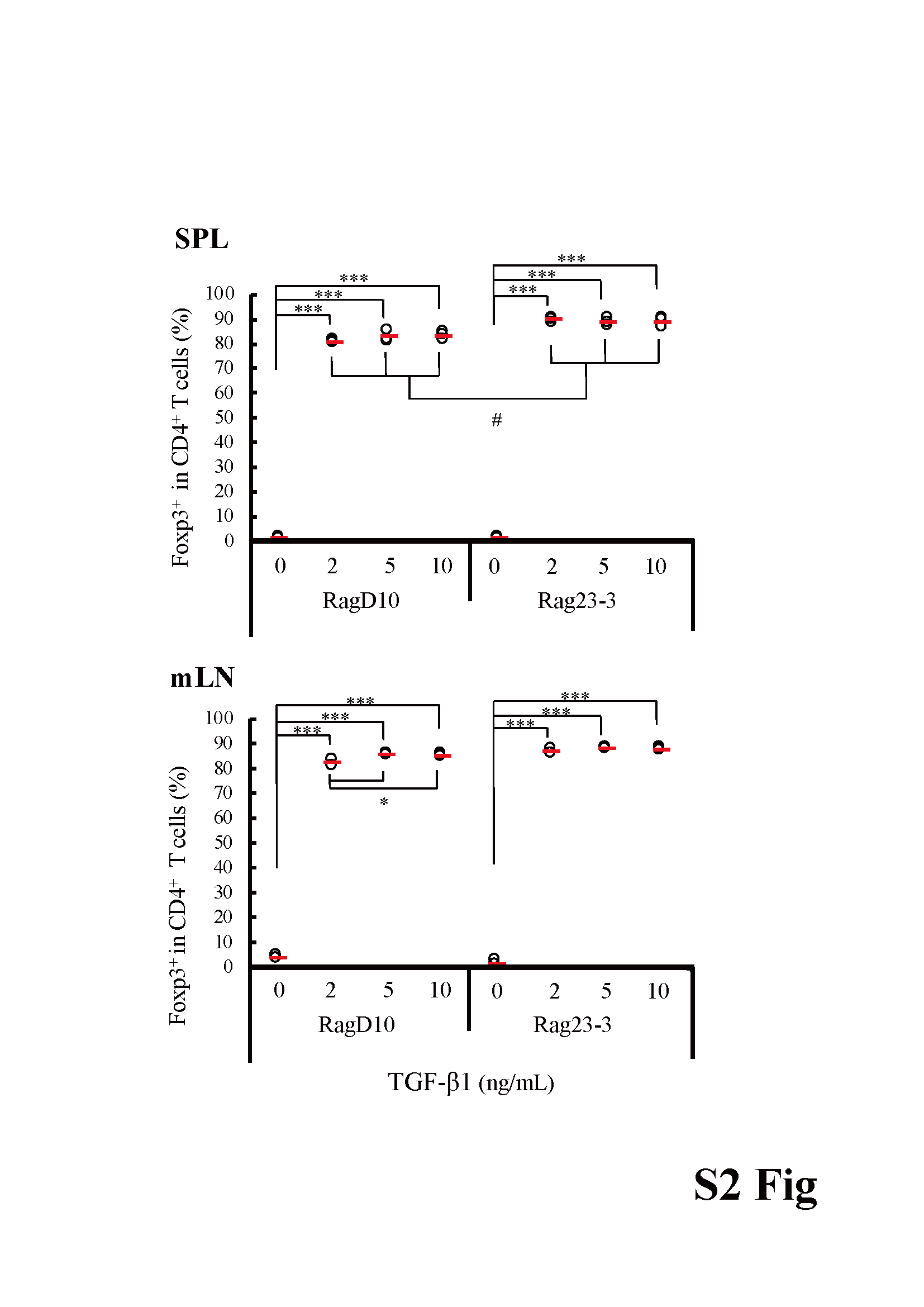

Supplement: S2 Fig — Spleen (SPL) and mesenteric lymph nodes (mLN) were harvested from untreated RagD10 or Rag23−3 mice, and CD4+ T cells were isolated by magnetic cell separation system. The cells were then stimulated with plate-bound anti-CD3 and anti-CD28 monoclonal antibodies and cultured in the presence of the indicated amounts of TGF-β1, retinoic acid (1 µM), and recombinant IL-2 (2 ng/mL) for 48 h, and the frequency of regulatory T cells (Foxp3+) within the CD4+ T cell population was determined. Each circle indicates the value for an individual well, and the horizontal lines indicate mean values (n = 3, mixture of cells from three mice/group). Data are representative of two independent experiments. Statistical analysis: Tukey’s HSD test [(#p < 0.05 (between different strains); * p < 0.05, ** p < 0.01, and *** p < 0.001 (between different groups in each strain)]. (TIFF) [file pone.0324105.s002.tiff]

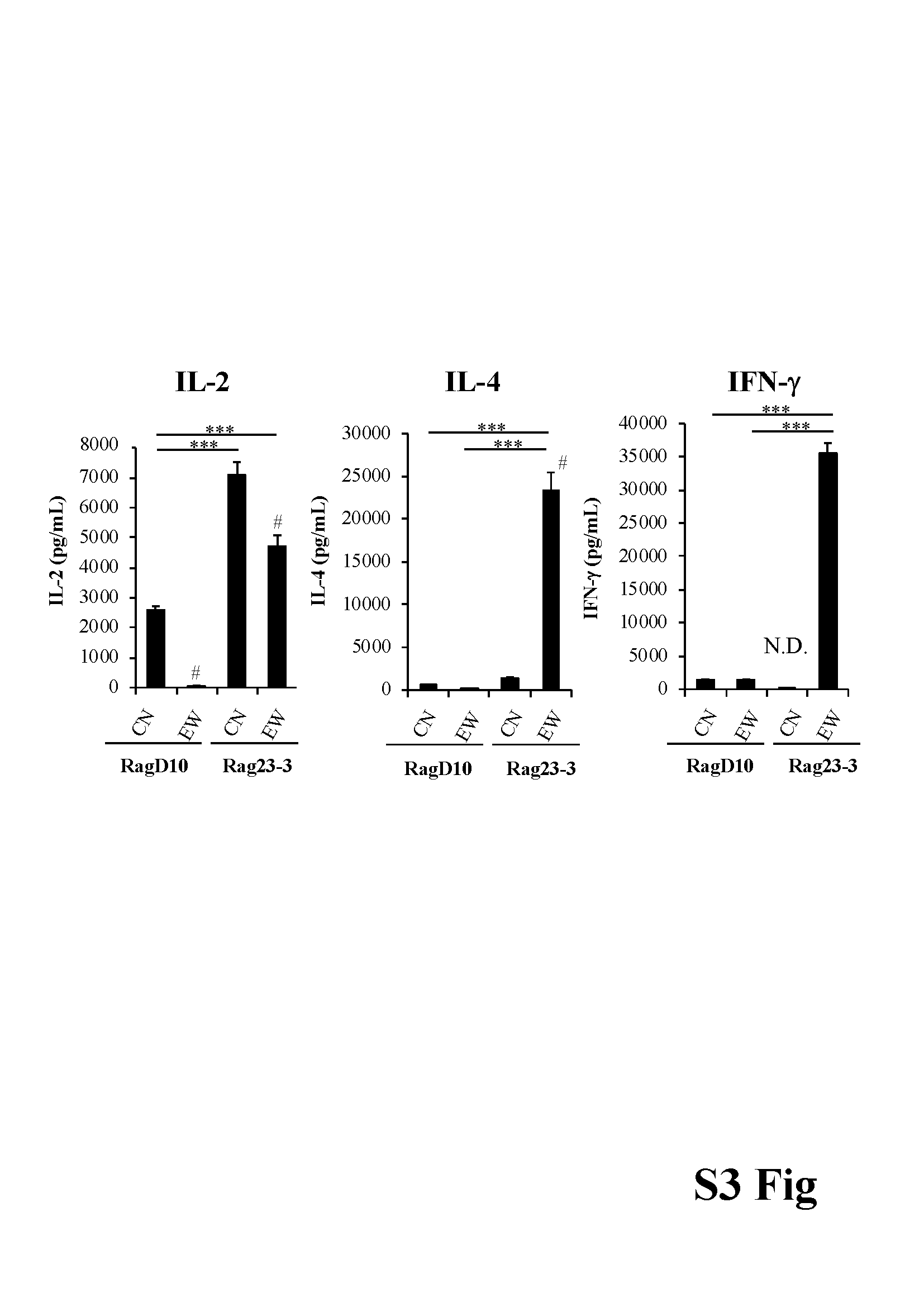

Supplement: S3 Fig — RagD10 and Rag23−3 mice were fed a diet containing egg white (EW) or casein (CN; control) for 7 days and spleens were harvested; CD4+ T cells were isolated, stimulated with plate-bound anti-CD3 and anti-CD28 monoclonal antibodies, and cultured under Treg polarization conditions for 48 h, and the culture supernatants were collected. The concentrations of interleukin (IL)-2, IL-4, and interferon gamma (IFN-γ) in the supernatants were determined by enzyme-linked immunosorbent assay (n = 3, mixture of cells from three mice/group). Data are representative of two independent experiments. Statistical analysis: Tukey’s HSD test [#p < 0.05 (CN vs EW in each strain); * p < 0.05, ** p < 0.01, and *** p < 0.001 (between different groups)]. N.D. = not detected. (TIFF) [file pone.0324105.s003.tiff]

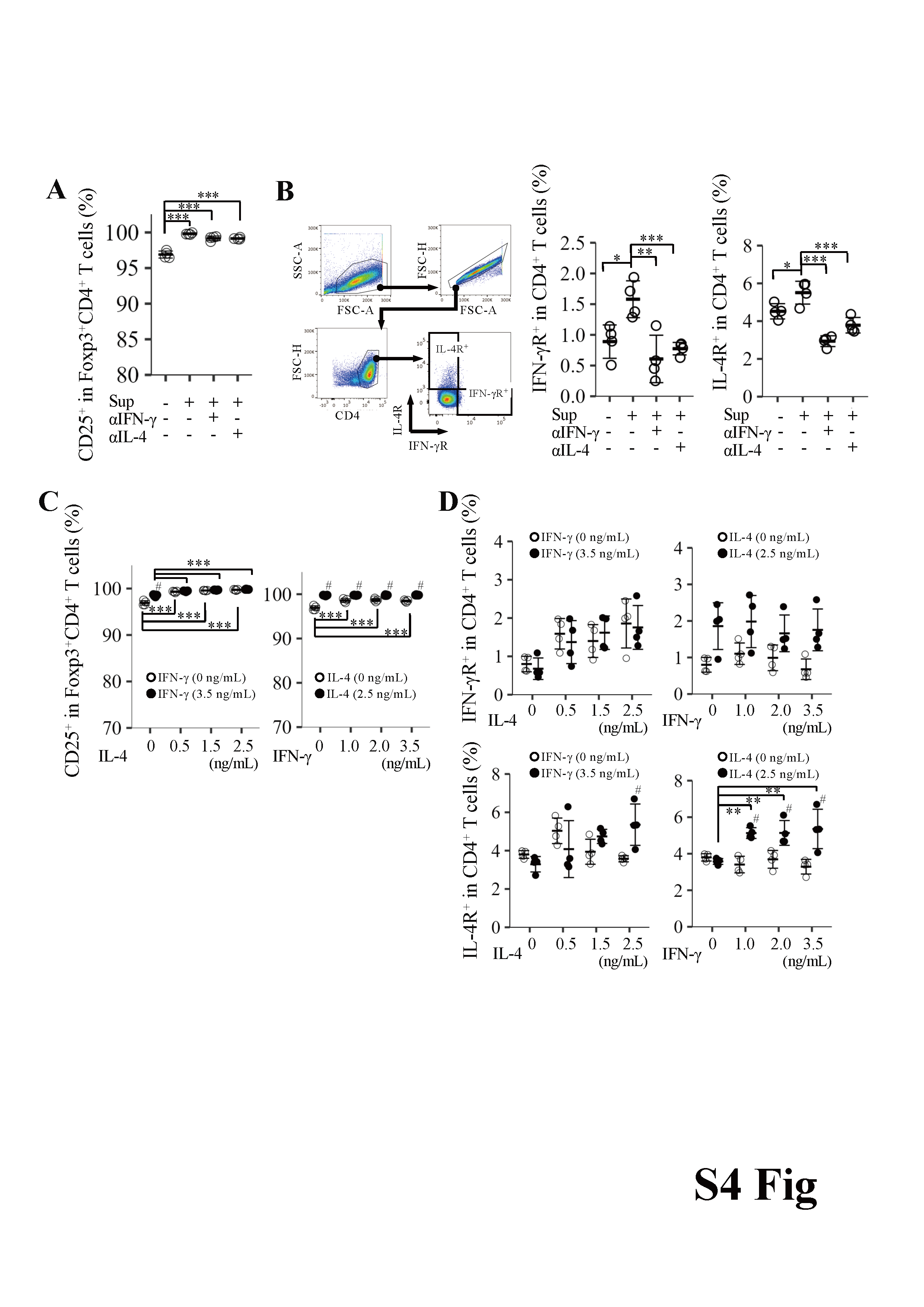

Supplement: S4 Fig — A) Frequency of CD25+ cells in regulatory T cells (Tregs; Foxp3+CD4+ T cells) differentiated from splenocytes of untreated-RagD10 mice cultured under Treg-polarization culture conditions described in the caption to Fig 3B. B) Gating strategy for identifying IFN-γ receptor (IFN-γR+) or IL-4 receptor (IL-4R+) on CD4+ T cells (left) and the frequencies of each receptor expression cells in CD4+ T cells (right). Each plot indicates the value for an individual well and horizontal lines indicate mean values (n = 3, mixture of cells from three mice/group). C) Frequency of CD25+ cells in Tregs from spleen cells of untreated RagD10 mice cultured under the Treg-polarization culture conditions indicated in Fig 3D and 3E. D) Frequencies of IFN-γR+ or IL-4R+ on CD4+ cells supplemented with rIL-4 (left: 0, 0.5, 1.5, 2.5 ng/mL) or rIFN-γ (right: 0, 1.0, 2.0, 3.5 ng/mL). Error bars indicate means ± SD (n = 4, mixture of cells from two to three mice/group). Analysis: Tukey’s HSD test {#p < 0.05 [IFN-γ (0 ng/mL)] vs [IFN-γ (3.5 ng/mL)] or [IL-4 (0 ng/mL)] vs [IL-4 (2.5 ng/mL)] in Fig S4C and S4D; * p < 0.05, ** p < 0.01, and *** p < 0.001 (between different groups)}. (TIFF) [file pone.0324105.s004.tiff]

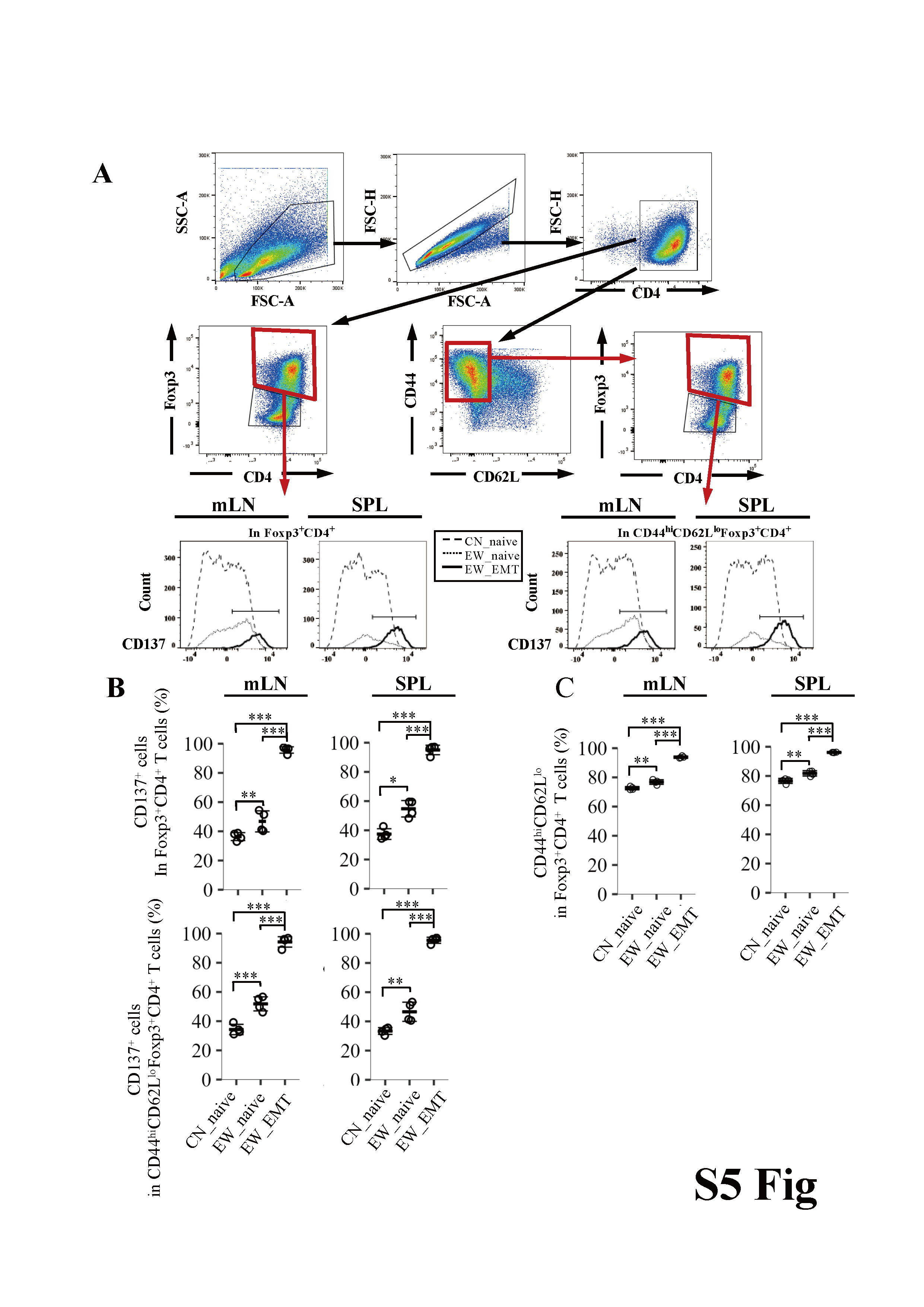

Supplement: S5 Fig — Naïve-like CD4+ T cells (CD44loCD62Lhi) and effector/effector memory CD4+ T cells (EMT; CD44hiCD62Llo) from Rag23−3 mice fed a diet containing egg white (EW) or casein (CN; control) for 7 days were stimulated with plate-bound anti-CD3 and anti-CD28 monoclonal antibodies and cultured under Treg-polarization culture conditions for 72 h. A) Gating strategy used to identify the CD137+ subpopulation within the Foxp3+ CD4+ and CD44hiCD62LloFoxp3+CD4+ populations. B) Frequency of CD137+ cells within the Foxp3+CD4+ and CD44hiCD62LloFoxp3+CD4+ T cell populations. C) Frequency of CD44hiCD62Llo cells within the Foxp3+CD4+ T cell population. In panels 5B and 5C, plots indicate the values for individual wells and horizontal lines indicate mean values (CN_naive: n = 4, EW_naive: n = 4, EW_EMT: n = 4, using a mixture of cells from 6 (CN) or 3 (EW) mice/group). Data are representative of two independent experiments. Statistical analysis: Tukey’s HSD test [*p < 0.05, ** p < 0.01, and *** p < 0.001 (between different groups)]. (TIFF) [file pone.0324105.s005.tiff]
