# Supplementary material for: Ovalbumin-specific regulatory T cells differentiated from the naïve phenotype (CD44loCD62Lhi) in mesenteric lymph nodes stably suppress enteropathy even in severe food-allergic mice
Source: PLoS One. 2025 May 30;20(5):e0324105. doi: 10.1371/journal.pone.0324105 (PMC12124522; doi:10.1371/journal.pone.0324105)
Supplement: Attachment — Submitted filename: Responses to Commentsfinal.pdf [file pone.0324105.s007.pdf]

Responses to the Editor's Comments

Comment 1

Please ensure that your manuscript meets PLOS ONE's style requirements, including those for file naming. The PLOS ONE style templates can be found at https://journals.plos.org/plosone/s/file?id=ba62/PLOSOne_formatting_sample_title_authors_affiliations.pdf and https://journals.plos.org/plosone/s/file?id=wjVg/PLOSOne_formatting_sample_main_body.pdf

Responses

As requested, we have formatted the manuscript in line with PLOS ONE's style requirements.

Comment 2

To comply with PLOS ONE submissions requirements, in your Methods section, please provide additional information regarding the experiments involving animals and ensure you have included details on (1) methods of sacrifice, (2) methods of anesthesia and/or analgesia, and (3) efforts to alleviate suffering.

Responses

As requested, we have added the following information regarding the experiments involving animals in Methods section:

> "When necessary for cellular and histological analysis, mice were euthanized by cervical dislocation by experts. During the adoptive transfer of Tregs, to facilitate the subsequent injection of cells into the caudal vein, a mouse was placed in a small box with only its tail sticking out through the hole in the box for a few minutes. The injection was performed without anesthetization as smoothly and quickly as possible by experts." (lines 147–152 in the revised manuscript)
>
> "Our experiments were approved without setting preemptive humane endpoints, because during the experimental period (7 days), the mice did not exhibit other clinical signs than weight loss." (lines 325–327 in the revised manuscript)

Comments 3 and 4

No. 3

We note that the grant information you provided in the 'Funding Information' and 'Financial Disclosure' sections do not match. When you resubmit, please ensure that you provide the correct

grant numbers for the awards you received for your study in the ‘Funding Information’ section.

Responses

We provided the correct grant numbers for the awards we received for our study in the “Funding Information” section in our corrected manuscript as follows:

> “This work was supported by grants from the Kieikai Research Foundation (HNA, Grant number; 2017S063, https://www.nakashima-foundation.org/kieikai/) and Grant-in-Aid for Scientific Research (B) from the Japan Society for the Promotion of Science (JSPS) (SH, Grant number; 26292065, https://www.jsps.go.jp/j-grantsinaid/index.html). The analysis for Fig 2a (CD25), Fig 3b–3g, Fig 5, S4 Fig, and S5 Fig was funded by Meiji Holdings Co., Ltd (HNA and SH), but we have no grant number assigned to this program grant in accordance with the nature of the grant, because The University of Tokyo's Corporate Sponsored Research Programs are programs established to conduct research on common issues that are of highly public nature in collaboration with the University of Tokyo, using funds received from the private sector and other external organizations. [Corporate Sponsored Research Programs | The University of Tokyo (u-tokyo.ac.jp)]”

No. 4

Thank you for stating the following financial disclosure:

[This work was supported by grants from the Kieikai Research Foundation (HNA, Grant number; 2017S063, https://www.nakashima-foundation.org/kieikai/) and
Grant-in-Aid for Scientific Research (B) from
the Japan Society for the Promotion of Science (JSPS) (SH, Grant number; 26292065, https://www.jsps.go.jp/j-grantsinaid/index.html) .
The analysis for Fig. 5 and S5 Fig was funded by Meiji Holdings Co., Ltd (HNA and SH).
Please state what role the funders took in the study. If the funders had no role, please state: ""The funders had no role in study design, data collection and analysis, decision to publish, or preparation of the manuscript.""
If this statement is not correct you must amend it as needed.
Please include this amended Role of Funder statement in your cover letter; we will change the online submission form on your behalf.

Responses

We have included the following Role of Funder statement in the cover letter to this response

document:

> "The funders had no role in the study design, data collection and analysis, decision to publish, or preparation of the manuscript."

In addition, in this revision, we have added several data obtained by some experiments. These experiments have been performed by the funding support by Meiji Holdings Co., Ltd. Thus, we would like to update this financial disclosure as follows:

> "This work was supported by grants from the Kieikai Research Foundation (HNA, Grant number; 2017S063, https://www.nakashima-foundation.org/kieikai/) and Grant-in-Aid for Scientific Research (B) from the Japan Society for the Promotion of Science (JSPS) (SH, Grant number; 26292065, https://www.jsps.go.jp/j-grantsinaid/index.html). The analysis for Fig 2a (CD25), Fig 3b–3g, Fig 5, S4 Fig, and S5 Fig was funded by Meiji Holdings Co., Ltd (HNA and SH), but we have no grant number assigned to this program grant in accordance with the nature of the grant, because The University of Tokyo's Corporate Sponsored Research Programs are programs established to conduct research on common issues that are of highly public nature in collaboration with the University of Tokyo, using funds received from the private sector and other external organizations. [Corporate Sponsored Research Programs | The University of Tokyo (u-tokyo.ac.jp)].
> The funders had no role in the study design, data collection and analysis, decision to publish, or preparation of the manuscript."

This update of financial disclosure is included in our cover letter.

Comment 5

Thank you for stating the following in the Competing Interests section:
[I have read the journal's policy and the authors of this manuscript have the following competing interests: [Meiji Holdings Co.,Ltd.].
Please confirm that this does not alter your adherence to all PLOS ONE policies on sharing data and materials, by including the following statement: ""This does not alter our adherence to PLOS ONE policies on sharing data and materials." (as detailed online in our guide for authors http://journals.plos.org/plosone/s/competing-interests). If there are restrictions on sharing of data and/or materials, please state these. Please note that we cannot proceed with consideration of your article until this information has been declared.

Please include your updated Competing Interests statement in your cover letter; we will change the online submission form on your behalf.

Response

We have added the following text to the Competing Interests section of our revised manuscript:

> "all of the authors of this manuscript had read the journal's policy and that the authors had a competent interest with Meiji Holdings Co., Ltd. This does not alter our adherence to PLOS ONE policies on sharing data and materials."

This statement is also declared in the cover letter to this response document.

Comment 6

We note that you have included the phrase "data not shown" in your manuscript. Unfortunately, this does not meet our data sharing requirements. PLOS does not permit references to inaccessible data. We require that authors provide all relevant data within the paper, Supporting Information files, or in an acceptable, public repository. Please add a citation to support this phrase or upload the data that corresponds with these findings to a stable repository (such as Figshare or Dryad) and provide and URLs, DOIs, or accession numbers that may be used to access these data. Or, if the data are not a core part of the research being presented in your study, we ask that you remove the phrase that refers to these data.

Response

As requested, we have removed the phrase "data not shown" from the revised manuscript. In addition, we have corrected the citation for Fig 2c to Fig 2b and now cite S3 Fig (corrected S4 Fig) as shown in line 496–497 in the revised manuscript as follows:

> "The cytokine profiles of the supernatants used in this experiment are shown in S3 Fig, and these profiles were consistent with those shown in Fig 2b."

Comment 7

Please include captions for your Supporting Information files at the end of your manuscript, and update any intext citations to match accordingly. Please see our Supporting Information guidelines for more information: http://journals.plos.org/plosone/s/supporting-information.

Response

As requested, we have added captions for our Supporting Information files (lines 1093−1158) at the end of our revised manuscript meeting PLOS ONE's style requirements.

Comment 8

Thank you for submitting manuscript. This research is very interesting, however, this manuscript is open to discussion in this time. As Reviewer indicated, title is too difficult to understand the contents of this manuscript. To be honest, this manuscript is hard to understand in whole. So, this manuscript needs to help with English language editing. As major point, this study just focused on the difference of R23-3 and RagD10, and the experiments and discussion were lack for the development of clinical application. Thus, this manuscript requires significant revision.

Response

We appreciate the opportunity to revise and resubmit our manuscript. We have updated the title to more simply describe our study to readers. In addition, the English in the revised manuscript has been edited by two native-English-speaking professional scientific editors from ELSS, Inc. (http://www.elss.co.jp), who also provided suggestions on ways to improve the clarity of the revised manuscript and according to the suggestion, the original manuscript have been throughout updated. In the Discussion, we now discuss the clinical application (line 889–899 in the revised manuscript). The remaining changes that we have made in response to the Reviewer’s comments are explained on the following pages. In addition, to describe the possibility of clinical application of treatment of Treg-transfer in the intestinal diseases, we added (ref. 15: *Int J Mol Sci*. 2020;21:7015).

Responses to Reviewer 1

Comment 1

The title is difficult to understand the aim of this study.

Response

We have updated the title as follows:

> Old title: "Naïve intestinal T cells in food-allergic model mice, expanded with anti-CD3 and anti-CD28 antibodies while under regulatory T cell-polarization culture condition, can exhibit suppressive activity"
>
> New title: "Ovalbumin-specific regulatory T cells with the naïve phenotype ($CD62L^{lo}CD44^{hi}$) from mesenteric lymph nodes suppress food-allergic enteropathy in mice, when expanded by anti-CD3/CD28 antibodies but not by ovalbumin plus antigen-presenting cells"

Comment 2

I do not understand the reason why the authors stimulated T cells by anti-CD3 and anti-CD28 antibodies. What does the stimulation with the antibodies physiologically mean?

Response

First, we observed that T cells from Rag23-3 mice have lower ability to differentiate into $Foxp3^{+}$ regulatory T cells (Tregs) than T cells from RagD10 mice. However, to promote T cell differentiation into Tregs, we needed an approach for stimulating and expanding the T cells. In addition, in performing an adoptive transfer of Tregs as a treatment, we have to differentiate and expand $CD4^{+}$ T cells into Tregs and to generate "poly-clonal Tregs" *in vitro* after isolating T cells from the individuals. If we used an antigen for the expansion in the culture, we would have to remove both the antigen-presenting cells and the antigen from the culture to increase the purity of the Tregs and to prevent side effects caused by contamination with the antigen. In addition, it has been reported that the functions and phenotype of Tregs differ depending on whether their differentiation was induced by using an antigen plus antigen-presenting cells or by using a combination of anti-CD3 and anti-CD28 antibodies (ref. 24: *Cellular Immunol*: 2014;290:179–184). Thus, in the present study, we examined the stimulation of T cell differentiation into Tregs by using anti-CD3 and anti-CD28 antibodies as well as OVA plus antigen-presenting cells. We now describe this rationale and a comparison of the data obtained with both approaches (Fig 1: antigen/antigen-presenting cell approach; Fig 2: antibody approach) in the revised Introduction

(line 113–119), Discussion (lines 769–788) and at the beginning of the Results (lines 422–427). See also our response to Comment 3.

Comment 3

Related to above comment, I do not understand the reason why the responses to anti-CD3 and anti-CD28 were different from that to antigen stimulation.

Response

As Reviewer 1 indicates, we did not clarify in our original manuscript the reasons for the different outcomes when Treg differentiation is induced by using the antigen/antigen-presenting cell approach or the antibody approach. As mentioned in our response to the previous comment, there is a report showing that there are functional and phenotypic differences between the iTreg lineages induced using either an antigen (HEL)/antigen-presenting cell approach or the combination of anti-CD3/anti-CD28 antibodies (ref. 24: *Cellular Immunol.* 2014;290:179–184). Therefore, our result has a precedent. However, considering together the present and previous comments from the Reviewer, we have added the following sentences to the revised Discussion to explain the different outcomes from the different approaches to induce Treg differentiation (lines 769–788); the reference mentioned in our responses to comments 2 and 3 has also been added to the revised manuscript as reference 24:

> "Although in the present study we found that T cells from EW-fed Rag23-3 mice have lower ability to differentiate into Tregs than T cells from RagD10 mice, we also found that using a combination of anti-CD3 and anti-CD28 mAbs to obtain sufficient volume of Tregs for adoptive transfer to other mice was superior to using a combination of antigen (OVA) and antigen-presenting cells. Although we did not investigate in detail why the responses to the Ab-stimulation were different from those to the antigen stimulation, similar findings have been reported elsewhere: Zhao et al. reported that the inhibitory effects on Th1 responses, surface molecules, and levels of Foxp3 expression in hen-egg-lysozyme–specific Tregs induced by mAbs are not the same as those induced by a combination of antigen plus antigen-presenting cells [24]. The implication of this when considering adoptive Treg transfer as a therapeutic option is that we have to choose the most appropriate approach to obtain Tregs with sufficient suppressive function. Our present results show that using mAbs to stimulate Treg differentiation affords Tregs with superior suppressive function in the context of severe food allergy. In addition, if we were to use the combination of antigen and antigen-presenting cells, we would also have to deal with removing the antigen and antigen-presenting cells from the culture at the end of the process to increase the purity of Tregs and

> avoid any potentially harmful effects of leftover antigen. Thus, the present findings highlight the potential of using Tregs differentiated from intestinal naïve T cells using a combination of anti-CD3 and anti-CD28 mAbs to suppress severe food allergic inflammation."

In addition, in the Introduction, we now clearly indicate the reason for using antibody-mediated stimulation rather than antigen-mediated stimulation as follows (lines 113–119 in the revised manuscript):

> "It has been reported that the functions and phenotypes of Tregs differentiated by using a combination of anti-CD3 and anti-CD28 monoclonal antibodies (mAbs) differ from those of Tregs differentiated with an antigen in the presence of antigen-presenting cells [24]. Thus, in the present study, we examined how to obtain stable suppressive Tregs from the Rag23-3 food-allergic enteropathy model by comparing both the inducing means and their resultant differentiation levels and functions of Tregs induced in Rag23-3 and RagD10 mice when fed EW."

Comment 4

The authors mentioned the Tregs from the mice as 'allergen-specific Tregs". I was wondering if the Tregs inhibited allergy in an allergen-specific manner. The authors should confirm the allergen specificity of the suppressive activity.

Response

Both the Rag23-3 and RagD10 mice used in our study produce only OVA-specific T cells in response to OVA stimulation, as was described in the Discussion in the original (lines 615–618). However, we agree with Reviewer 2 that this is important information. Thus, we have therefore added the following additional description to "Materials and Method, Animals" (lines 136–139 in the revised manuscript):

> "The only lymphocytes that these strains of mice produce in response to OVA challenge are OVA-specific T cells; therefore, we can observe the antigen-specific T cell responses without consideration of B cell responses."

Comment 5

The authors wrote "Naïve CD4+ T cells isolated from CN-fed Rag23-3 mice and stimulated with anti-CD3 and anti-CD28 mAbs differentiate into Tregs" (line 369). However, the authors used not only CD-fed mice but also EW-fed mice in the section. I do not understand the reason why

title of the section was different from the content.

Response

In response to the Reviewer's comment, we have updated the title of Fig 2 (line 369) as follows:

> Old title: "Naïve $CD4^+$ T cells isolated from CN-fed Rag23-3 mice and stimulated with anti-CD3 and anti-CD28 mAbs differentiate into Tregs"
>
> New title (lines 419–421): "Antibodies stimulation induces $CD4^+$ T cells from CN-fed, but not EW-fed Rag23-3 mice to differentiate into Tregs, whereas $CD4^+$ T cells from RagD10 mice differentiate regardless of diet"

Comment 6

The position of markers in the graph right bottom of Fig. 2a seems out of alignment.

Response

We have corrected the mark positions in Fig 2 out of alignment (Fig 2 revise). In addition, by adding the panels of the frequency of $CD25^+$ in $Foxp3^+CD4^+$ T cells, we moved the position of panels of frequencies of EMT next to that of Tregs.

Comment 7

The authors wrote "Fig. 2c (data not shown)." (line 434). The authors should provide all data you used in this manuscript.

Response

This was the result of our misnumbering the figure, as indicated in our response against Editor, Comment 6. We have corrected the number, added an additional figure citation, and removed "data not shown".

Comment 8

Fig. 3b shows that each of the anti-IL-4 or anti-IFN-g antibody almost completely inhibited the activity of the Sup by itself. I was wondering why IFN-g in anti-IL-4 condition or IL-4 in anti-IFN-g condition did not affect Treg induction.

Response

To address the Reviewer's concern, we have performed several experiments analyzing

concentration of IL-4 or IFN-gamma in the supernatant by ELISA, and the expression of IL-4 receptor or IFN-gamma receptor, frequencies of regulatory T cells (Tregs) or effector/effector memory T cells (EMT) in the cells cultured under Treg polarization condition with/without recombinant IL-4 or IFN-gamma (lines 263–269) by Flow cytometry (lines 222–224). We have added the data to both Fig 3 and a figure newly created as S4 Fig. The data are also described in the revised Results (lines 510–565) and Discussion (lines 846–888) sections.

As a result, we found the mechanism regulating Treg differentiation under severe allergic circumstances: On the start of feeing EW to Rag23-3 mice, a greater amount of IL-4 might be strongly produced by EMTs, which would promote the differentiation of T cells into EMTs producing not only IL-4 but IFN-gamma overproduction. However, IL-4 only was associated with EMT activation. One of the cytokines affected production of the other cytokine regulating their receptors expression. Overproduction of IFN-gamma further promoted IL-4 production, creating a feedback loop that works to balance the effects of the two cytokines. Both cytokines directly or through the loop leaded to inhibition of Treg differentiation. Our data indicated in the revised manuscript showed that by this neutralization with each antibody, either cytokine concentration was decreased, EMT activation was subsided, and Treg differentiation was enhanced. To describe and discuss the results, we have added a reference (ref 45: *Sci Rep*.2021;11:1045). Unfortunately, given the complexity of these experiments, their inclusion has somewhat lengthened our manuscript, especially the Results; we hope that this is okay.

Comment 9

I was wondering why the characteristics of nsïve T cells of EW-fed mice differed from those of CN-fed mice in Fig. 5c. Were the naïve T cells of EW-fed mice really naïve?]

Response

We agree with the Reviewer that this is an important finding from the experiment shown in Fig 5. We think that the naïve T cells from the EW-fed mice might be one of several lineages of naïve T cells. However, when we purified naïve T cells as cells having $CD62L^{lo}CD44^{hi}CD4^{+}$ phenotype using a magnetic-activated cell sorting system or FACSAria II cell sorter, we got similar results with both approaches. Although we did not clarify whether the naïve T cells from the EW-fed mice really were naïve, the manufacturer's instructions for the magnetic-activated cell sorting system we used to obtain the naïve T cells (Miltenyi Biotec, Bergisch Gladbach, Germany) defined the naïve phenotype as $CD62L^{lo}CD44^{hi}CD4^{+}$. In light of the absence of data to prove that the cells were naïve, to address the Reviewer's concern we have updated the description of "naïve" in the caption for Fig 5 and the parts of the text related to the discussion of the naïve T cells of EW-fed mice to use the term "naïve-like cells" (highlighted in yellow in the revised manuscript).

In addition, this description of the word "naïve-like" was also added to Result section of our revised manuscript (lines 681-683)

Comment 10

It was very difficult to understand the purpose of each experiment throughout the manuscript. Please give it a clear title easy to understand the purpose of each section of results and explain the reason why the experiments were adopted for the purpose of each section.

Response

We have updated the titles of the subsections in the Result section to clarify the meaning and allow readers to easily understand the purpose of each part of the study. The changes as follows:

Fig 1 (lines 344–346 in the revised manuscript)

Old title: "Stimulation with OVA impaired the induction of Tregs, but promoted the differentiation of effector/effector memory T (EMT) cells under a characteristic cytokine milieu in Rag23-3 mice"

New title: "Stimulation with OVA impairs Treg induction but promotes EMT induction and IL-4 and IFN-gamma production in Rag23-3 mice, but not in RagD10 mice"

In addition, the last sentence of the section in the original manuscript (lines 345–348) was moved to the beginning of the next section (line 425 in the revised manuscript) and corrected from "non-TCR-mediated (non-OVA)" to "no-antigen-mediated" to avoid misleading readers regarding the approach using the anti-CD3 and anti-CD28 antibodies.

Fig 2 (lines 419–421 in the revised manuscript)

Old title: "Naïve $CD4^+$ T cells isolated from CN-fed Rag23-3 mice and stimulated with anti-CD3 and anti-CD28 mAbs differentiate into Tregs"

New title: "Antibodies stimulation induces $CD4^+$ T cells from CN-fed, but not EW-fed Rag23-3 mice to differentiate into Tregs, whereas $CD4^+$ T cells from RagD10 mice differentiate regardless of diet"

Fig 3 (lines 487–489 in the revised manuscript)

Old title: "Overproduction of IL-4 and IFN-gamma inhibit Treg differentiation in EW-fed Rag23-3 mice"

New title: “Excessive IL-4 and IFN-gamma production by $CD4^+$ T cells from EW-fed Rag23-3 mice suppresses Treg differentiation of $CD4^+$ T cells from RagD10 mice

To clearly describe the aim of this study and the adoption of the experiments, we have moved the following sentence (line 406–408 in the original manuscript).

> “In EW-fed Rag23-3 mice, the higher frequency of EMT cells occurred concomitantly with greater cytokine production and appeared to lead to $CD4^+$ T cells not differentiating into Tregs.”

to later in the text (lines 490–496 in the revised manuscript) and updated it as follows:

> “Unlike in EW-fed RagD10 mice, the higher frequency of EMT induction in the EW-fed Rag23-3 mice was observed to have occurred concomitantly with greater cytokine production, suggesting that this higher cytokine production prevented the differentiation of $CD4^+$ T cells into Tregs. To examine this further, we first examined whether the supernatants of $CD4^+$ T cells isolated from the spleen of EW- or CN-fed Rag23-3 or RagD10 mice did indeed have the ability to prevent the differentiation of $CD4^+$ T cells into Tregs.”

Fig 4 (lines 595–597 in the revised manuscript)

> Old title: “Tregs derived from Rag23-3 mice have sufficient regulatory activity to prevent allergic inflammation”
>
> New title: “Adoptive transfer of Ab-stimulated Tregs from untreated Rag23-3 mice to EW-fed Rag23-3 mice have sufficient regulatory activity to prevent allergic inflammation”

In addition, in the beginning of the section, we added the sentence in the revised manuscript (lines 598–601):

> “We proved in Fig 2 that naïve $CD4^+$ T cells had similar ability to differentiate into Tregs in Rag23-3 mice compared with those in RagD10 mice. Therefore, we next investigated whether the Tregs induced from Rag23-3 $CD4^+$ T cells possess sufficient regulatory activity to inhibit intestinal allergic responses.”

Fig 5 (lines 647–649 in the revised manuscript);
The title of this section was not changed, but we have updated the sentence at the beginning of the section (lines 650–656 in the revised manuscript) from

> "We have shown that $CD4^+$ T cells of untreated Rag23-3 mice have the potential to differentiate into Tregs (Fig 2), so we next examined if the naive phenotype $CD4^+$ T cells in Rag23-3 mice with enteropathy also keep their ability to differentiate into Tregs in response to stimulation by anti-CD3 and anti-CD28 mAbs"

to

> "We have shown that $CD4^+$ T cells of CN-fed Rag23-3 mice have the potential to differentiate into Tregs (Fig 2) when they are stimulated and expanded with anti-CD3 and anti-CD28 mAbs, but it was unclear whether the naïve $CD4^+$ T cells in EW-fed Rag23-3 mice with enteropathy also keep their ability to differentiate into Tregs. Therefore, we next examined if the naive $CD4^+$ T cells purified from the spleens or mLNs cells isolated from EW-fed Rag23-3 mice can differentiate into Tregs when they were stimulated with anti-CD3 and anti-CD28 mAbs."

Responses to Reviewer 2

Base

Although the length of manuscript is not limited in PONE journal, the text in method, result and figure legends is overlapping, that makes the redundant impression to the readers. Especially, the text in the figure legends may be shorter, because they use almost an equal volume as the text.

Response

We have shortened the legends to Figs 1–5 and removed overlapping descriptions in the Results section and Methods sections.

Comment 1

The ref. 1, 2, 4 should be updated to represent the recent clinical situations regarding food allergy and oral immunotherapy.

Response

We have updated refs. 1, 2, and 4 (corrected to 5) in our corrected manuscript as follows:

1) *Pediatr Allergy Immunol.* 2021;32:647-657. https://doi: 10.1111/pai.13443.

2) *J Allergy Clin Immunol.* 2018;141:41-58. https://doi: 10.1016/j.jaci.2017.11.003.

5) *Allergy.* 2020;75:1337-1346. https://doi: 10.1111/all.14220.

In addition, we have a new ref. 3:

3) *Cellular Immunol.* 2023;383:104650. https://doi.org/10.1016/j.cellimm.2022.104650.

With this addition, ref. 3 in the original manuscript has been renumbered to ref. 4 in the revised manuscript.

Comment 2

L. 371-373 Immobilized anti-CD3 stimulation should be TCR-mediated, although not antigen-specific stimulation.

Response

As described in the response to Reviewer1, Comment 10, we have corrected the term “non-TCR-mediated (non-OVA)” to “no-antigen-mediated to avoid any misunderstanding regarding the approach using the anti-CD3 and anti-CD28 antibodies.

Comment 3

L. 434 Fig 2c might be Fig 2b?

Response

We have corrected the incorrect figure citation from Fig 2c (line 467) to Fig 2b. In addition, to address Editor, Comment 6, the following sentence was also corrected now citing S3 Fig (corrected S4 Fig) as shown in lines 496–497 in the revised manuscript as follows:

> “The cytokine profiles of the supernatants used in this experiment are shown in S3 Fig, and these profiles were consistent with those shown in Fig 2b.”

Comment 4

Statement of S3 Fig is missing in the text.

Response

In the revised manuscript, we have deleted the statement related to S3 Fig and S3 Fig itself, that had too much information for the readers and did not clearly describe the aim of this study. Then, we think that the deletion would be better for our revised manuscript to be concise.

Comment 5

L.439-442 Blocking either IL-4 or IFN-gamma alone almost completely restored the suppressive effect of the Rag23-3_EW_Sup, suggesting these two cytokines are affecting to one lymphocyte simultaneously. How do authors explain this mechanism?

Response

Please see our response to Reviewer 1, Comment 8.

Comment 6

L. 658-660 In this TCR-transgenic mouse model, how authors consider the difference between OVA+APC stimulation and anti-CD3+anti-CD28 stimulation? The difference of TCR signal transduction, or the other signals through co-stimulatory molecules on the APC? In other words, does OVA+APC stimulation with Treg-polarization condition on EW-fed Rag23-3 cells have a potential to induce Tregs?

Response

Previously, we reported that prolonged EW-feeding from 7 to 28 days induced the differentiation

of $Foxp3^+CD4^+$ T cells from naïve cells in EW-fed Rag23-3 mice, in which the T-cell receptor was activated in the presence of an antigen (ovalbumin) and antigen-presenting cells (ref. 22 in the revised manuscript). From this we concluded that naïve $CD4^+$ T cells of EW-fed Rag23-3 have the potential to differentiate Tregs, and this was described in our original manuscript (line103–107). However, we understand that our description may not have been clear enough. Please see our explanation about the differences between the antigen- and antibody-mediated approaches to stimulate Treg differentiation in our response to Reviewer 1, Comment 3 and the associated changes we have made to the manuscript.

Responses to Reviewer 3

Comment 1

This manuscript is difficult to follow and understand the emphasized points in its current version. The manuscript could benefit from English language editing to enhance clarity and readability. I strongly suggest this is necessary in its current format.

Response

To address the Reviewer's concern, the English in the revised manuscript has been edited by two native-English-speaking professional scientific editors from ELSS, Inc. (http://www.elss.co.jp), who also provided suggestions on ways to improve the clarity of the revised manuscript.

Comment 2

The study begins with the introduction of potential therapy using Treg administration for food allergy patients. Is there any clinical perspective on that? To avoid confusion for the reader, the authors should cite adequate references for Treg therapy in food allergies. Compared to other inflammatory diseases, cellular interventions such as cell therapy for food allergies represent an ultimate approach. The importance of how this research can be substantiated is in question.

Response

The literature does not yet contain adequate references for the use of Treg therapy for the treatment of food allergies. This lack of studies may be because the contribution of Tregs to oral immunotherapy or natural recovery remains unclear, although Tregs and their function mediated through inhibitory cytokines have been clarified in the natural resolution of food allergy (ref. 3: *Cellular Immunol.* 2023: 383; 104650) and in the analysis of allergen-specific Tregs in oral immunotherapy (ref. 2: *J Allergy Clin Immunol.* 2018;141:1699-1710 and ref. 16: *Front Immunol.* 2021;12:705615. However, the therapeutic potential of Foxp3^{+} T cell transfer has been described in allergy as well as in several autoimmune diseases (ref. 37: *Front Immunol.* 2022;13:956907.). Therefore, we think that Treg transfer is a potential treatment approach for the treatment of persistent food allergy, for which there currently no effective treatment. Therefore, even if we are unable to provide the adequate references related to Treg therapy for food allergies in our present manuscript, publication of our findings in PLOS ONE are sure to contribute to future progress in the treatment of severe food allergy. We agree with the Reviewer3 that the report must be written in a manner that avoids confusing readers; we have added the following sentences explaining the rationale described above to the revised manuscript, which includes two new references, as

explained above (ref 3: *Cellular Immunol.* 2023;383:104650 and ref 37: *Front Immunol.* 2022;13:956907. lines 888–898):

> "Adoptive transfer of Tregs for the treatment of severe inflammatory diseases is an attractive approach because it has the potential to not only treat but also prevent severe inflammatory diseases including allergy [3], although there have not yet been any clinical trials of Treg transfer therapy. There are, however, currently three approaches that have been reported to obtain stable Tregs from peripheral blood mononuclear cells: naturally occurring regulatory T cells (nTregs) [46], chimeric antigen receptor regulatory T cells (CAR-Tregs) [47], and stable and functional induced regulatory T cells (S/F-iTregs) [48]. It has been clarified in individuals who have outgrown their food allergy naturally or by OIT that Tregs and mediation of their function through inhibitory cytokines play important roles [3]. Therefore, Treg transfer may be a suitable approach for the treatment of individuals with persistent food allergy."

<u>Comment 3</u>

Two strains of OVA-specific TCR tg mice are used in the manuscript, and they published the differential function of Tregs between these mouse strains in a 2017 PLOS One paper. In that paper, the amount of IL-4 was shown to be a causative factor in the regulation of Treg function. In this manuscript, their aim is to understand the stability of Tregs, but the study lacks analysis of how the cells are "stabilized", for instance, how epigenetic regulations differ in the two mice lines of iTregs. How about RORgt+ Treg which is important cell population regulating allergic disorder. Is this caused by intrinsic control based on the strength of TCR signaling? How are Tregs differently induced in the two lines? The authors can experimentally define this by measuring the strength of TCR signaling.

Response

In the present study, we did not directly examine the stability of the Tregs. However, as noted by the Reviewer3, in our 2017 PLOS ONE paper, we did show a difference in the stability of the tolerance acquired in the two mouse strains by analyzing the recurrence of weight loss induction following a 1-month interval during which feeding was switched to a control diet without antigen. In response to the Reviewer3's comment, this result suggesting a lower stability of Tregs differentiated in EW-fed Rag23-3 mice compared with those in RagD10 mice has been added to the Introduction section in the revised manuscript (lines 106-112).

In our previous study, the strength of the T cell-receptor signal was investigated by examining proliferation and IL-4 or IFN-gamma production abilities of $CD4^{+}$ T cells stimulated

with ovalbumin plus antigen-presenting cells or with anti-CD3 antibody (ref 26: *Biosci. Biotechnol. Biochem*. 2012;76:1979-1981). These results were discussed in the original manuscript (lines 615–641) and they remain in the revised manuscript (lines 737−767). Regulation of Treg differentiation under an excessive cytokine milieu is now discussed in the revised manuscript and the data are shown in Fig 3 and S4 Fig (see our response to Reviewer1, Comment 8).

As Reviewer3 notes, RORgt+ Tregs in the intestine are an important cell population regulating food allergy. However, the purpose of the present study was not to elucidate the changes of the various Treg subtypes, rather it was to show a way to obtain stable Tregs under circumstances of severe food allergy. To address the Reviewer's point, we have added a reference that discusses the importance of RORgt^{+} Tregs in food allergy to the Discussion section of the revised manuscript (ref 39: *Immunity*. 2020;53:277-289)(lines 831):

"ROR-gamma t Tregs also may be one of the functional population [20, 39]."

Comment 4

Counter gating should be shown in Figure 1a of effector memory T cells with adequate control.

Response

We are sorry but we do not fully understand the meaning of "counter gating" in the context of our study. However, we have reviewed the gating strategy of whole cells and recalculated the resulting values. As a result, we have been able to correct the frequency of effector/effector memory T cells (EMTs). Thus, we have updated Fig 1a and 1b and added the frequency of CD25^{+} cells in the Tregs as Fig 1c (see also our response to Reviewer3, Comment 5). Although, the recalculated values have been added to the revised manuscript (lines 347–362), the findings have remained unchanged.

Reviewer3, Comment 7 also notes the high frequencies of EMTs in the casein-fed mice reported in the previous paper. However, our recalculation here revealed that the percentage of EMTs was only around 5%. Therefore, we have removed the associated sentences from the Results section of the revised manuscript (lines 316–318 in the original manuscript).

Comment 5

Upon activation, CD4^{+} T cells transiently express FOXP3, especially in activated CD4^{+}CD25^{-} T cells, thus CD25 expression should be shown in the panel throughout the manuscript.

Response

We agree that it would be advantageous to show CD25 expression data. To show that the Tregs

used in our study were almost all $CD25^+$, we have added data showing the frequency of expression of $CD25^+$ within regulatory T cell population ($Foxp3^+CD4^+$ T cells; Tregs) as Fig 1b, Fig 2a, and S4 Fig plus corresponding legends for each figure in the revised manuscript. Furthermore, we have added the following sentences in the Results section:

> lines 358–362
> "In both treatment groups for both mouse strains, around 80% of the Tregs expressed CD25, a common marker of murine Tregs and a component of the Treg IL-2 receptor (Fig 1b). The frequency of $CD25^+$ T cells significantly increased in EW-fed mice in spite of the difference of the strains, showing that EW feeding induced Tregs, even at minor level under the severe allergic condition of Rag23-3 mice."
>
> lines 444–446
> "In the present study, almost 100% of the induced Tregs in both models and with both diets expressed CD25 (Fig 2a), which is similar to what was observed for Tregs induced from mLN cells by antigen stimulation (Fig 1b)"
>
> Lines 517–518
> "Almost Tregs induced were $CD25^+$ (S4a Fig)."
>
> Lines 534–536
> "The Tregs induced were almost $CD25^+$ independently of the concentration of rIL-4 and/or IFN-gamma (S4c Fig)"

Finally, to add the frequency data to the figures, we have changed the presentation of the experimental groups in Figs 1 and 2 such that the casein-fed group is now designated by a white circle or bar and the egg white–fed group is designated by a black circle or black bar for both the Rag23-3 and RagD10 mice.

<u>Comment 6</u>

I don't understand why IL-2 is significantly induced in the control of Rag23-3 mice. Fully discuss this.

Response

The Reviewer notes that IL-2 production was significantly higher in the control of Rag23-3 mice than that in the control group of RagD10 mice, which is a similar result with our previous results

published in 2017 (ref 22, PLOS ONE. 2017;12:e0172795). As indicated in our previous report, the ability of $CD4^+$ T cells from Rag23-3 mice to proliferate in response to stimulation by ovalbumin is also stronger than that in RagD10 mice, suggesting that these cells can produce large amounts of IL-2 as a T cell proliferative factor, although we have not investigated the signal transduction in detail. To address the Reviewer's point, we have added the following sentence in the Result section of the revised manuscript (lines 374–376):

> "Taken together, these results indicate that OVA stimulation resulted in greater T cell proliferation in the Rag23-3 mice than in the RagD10 mice, which is consistent with our previous findings [22]."

In addition, we have added further discussion about the reason why the Treg population is very small in the CN-fed Rag23-3 mice (Fig 1b) despite high IL-2 production in the revised manuscript (lines 387–396), By adding this discussion, one reference was added (ref 28: *Immunity.* 2008;29:758-770).

Comment 7

TCR is specific for OVA; however, EMT cells are significantly induced in the mice without OVA. What is the underlying mechanism of these phenotypic discrepancies?

Response

Please see our response to Reviewer3, Comment 4.

Comment 8

The author indicated the importance of IL-4 for the induction of Treg. Does IL-4 administration induce Tregs in Rag23-3 CN mice?

Response

To confirm the ability of IL-4 to inhibit Treg differentiation, we performed the experiment described in our response to Reviewer2, Comment 8. We have added the results of this experiment as Fig 3 and discuss the findings in the Results (lines 510–565) and Discussion (lines 845–887) of the revised manuscript. The experiment was done by using RagD10 mice, but because Rag23-3 mice are more sensitive to the amount of excessive IL-4, we think that the data obtained by using RagD10 can address the Reviewer3's concern.

Comment 9

TGF-beta should be referred to as "TGF-beta1" throughout the manuscript.

Response

As requested we now use TGF beta1 throughout the revised manuscript.

Comment10

In terms of Treg induction via non-TCR mediated stimulation, I do not understand why the authors stimulated with an anti-CD3 antibody, which mimics TCR stimulation. The authors should reconsider the fundamental concept of this study and rearrange the experimental design.

Response

Upon reflection, we think that the use of "non-TCR-mediated (non-OVA)" was not appropriate for the context. As described in our response to Reviewer1, Comment 10 and Reviewer2, Comment 2, we have removed the expression "non-TCR-mediated (non-OVA)" and replaced it with "no-antigen-mediated" to more clearly describe the approach using anti-CD3 and anti-CD28 antibodies (Lines 422–427).

Comment 11

The authors stop explaining the details of the results obtained about the different induction rates of Tregs between spleen and MLN by citing the Mucosal Immunology paper from Traxinger BR. The authors should fully explain and conclude the results based on their data.

Response

In an oral tolerance model similar to RagD10 mice, we have reported that Foxp3 expression is more easily induced in the mesenteric lymph node (mLN) than in the spleen, but in a food allergic intestinal inflammation model like Rag23-3 or OVA23-3 mice, this is not necessarily the cases (ref. 22). This is because the $CD4^+$ T cells of the intestinal immune system (i.e., in the mesenteric lymph nodes) in food-allergy model mice produce high levels of IL-4 in response to ovalbumin that invades into the intestinal tract after eating the egg-white diet, so the differentiation of $CD4^+$ T cells into Tregs is prevented. However, in the spleen, the number of $CD4^+$ T cells and the level of IL-4 production are lower than those in mLN (ref. 22), which leads to higher induction of Tregs compared with that in the induction in the mLN. We have added the above description to the Results section in the revised manuscript (lines 437–444):

> "This is because $CD4^+$ T cells from the mLN in Rag23-3 mice produce high levels of IL-4 in response to OVA that invade into the intestinal tract after eating the EW diet, the

differentiation of $CD4^+$ T cells into Tregs, which is normally induced against antigen entry in the intestine indicated by Traxinger, et al. [30], is prevented [31]. In contrast, in the spleen, the number of $CD4^+$ T cells is less and the level of IL-4 production is lower than that in mLN [31], which leads to higher induction of Tregs compared with the induction in mLN."

Comment 12

IL-4 and IFNg are produced at higher levels in the Rag23-3 mice, thus Th1 and Th2 cells may be induced in the Treg-induction experiments. The authors should show if IL-4 and IFNg suppresses or skews Th1/Th2 cells by analyzing intracellular FACS of Th1/2 in mice.

Response

We have not been to conduct an intracellular flow cytometry analysis of Th1/2 cells. However, as described in our response to Reviewer1, Comment 8, we have performed additional flow cytometry experiments to examine the frequency of Tregs in $CD4^+$ T cells and of IL-4R- or IFN-gammaR expression in the Tregs, in which the Tregs were differentiated from $CD4^+$ T cells of untreated RagD10 mice under Treg polarization culture condition in the presence or absence of rIL-4 and/or rIFN-gamma. Please see our response to Reviewer1, Comment 8.

Comment 13

Line 403: "The finding that OVA did not fully induce Treg differentiation, but stimulation with antibodies induced Treg differentiation in CN-fed Rag23-3 mice suggests the presence of factors" –however, they mimic TCR signaling by anti-CD3 and anti-CD28. I don't understand this part.

Response

As described in our responses to Reviewer1, Comments 2 and 3, we have added the sentences explaining the difference in the T-cell-receptor signaling between cells induced stimulation by a combination of anti-CD3 and anti-CD28 antibodies or by a combination of ovalbumin and antigen-presenting cells. Please see our response to Reviewer1, Comment 2 and 3.

Comment 14

The authors indicate Tregs derived from Rag23-3 mice have sufficient regulatory activity; however, it is important to examine the functional differences in the suppressive roles of RagD10 and Rag23-3 derived Tregs in vivo and in vitro. Cytokine productions are different?

Response

As pointed out by the Reviewer3, we should describe in more detail the functional differences

between the Tregs from the two model strains used in our manuscript. In addition, we also think that the following aim, which was included in the original Introduction, did not do a good job of explaining our aims for the study (lines 108–110 in the original manuscript):

> "In the present study, we examined further how to obtain stable and suppressive Tregs by comparing the differentiation and function of Tregs between EW-fed RagD10 and Rag23-3 mice."

Thus, we have updated that part of the text in the revised manuscript as follows (lines 116–119):

> "Thus, in the present study, we examined how to obtain stable and suppressive Tregs from the Rag23-3 food-allergic enteropathy model by comparing both the inducing means and their resultant differentiation levels and functions of Tregs induced in Rag23-3 and RagD10 mice when fed EW"

We have not examined the differences in the functions of the Tregs from Rag23-3 and RagD10 mice at the molecular level; however, in our previous study, by analyzing the stability of Treg function induced by egg-white (EW) feeding for 28 days in both strains of mice (ref. 22) and the inhibitory effects of the Tregs on proliferative responses of effector/effector-memory T cells (EMTs) of Rag23-3 mice (currently under submission at another journal), we have data that show Tregs from EW-fed RagD10 mice are more stable than those of EW-fed Rag23-3 mice. Unfortunately, since our latter findings are currently under submission elsewhere, we are unable to add those data to the revised manuscript. Instead, we have added the following sentence in the revised manuscripts follows (lines 110–112):

> "Indeed, in the same study, we showed that the stability of OVA-specific Tregs induced during feeding with EW-diet was higher in DO11.10 mice than in OVA23-3 mice [22].

<u>Comment 15</u>

Lines 552 to 557 are hard to understand. Why do naïve T cells ameliorate differentiation to Tregs? This part is complicate to the reader. Please reorganize this part.

Response

We agree that the explanation of that point was insufficient. We have updated the text as follows:

<u>Old text (lines 552-557):</u>

“These results indicate that naïve phenotype $CD4^+$ T cells in the lymphoid tissues of Rag23-3 mice with severe allergic enteritis had reduced potential to differentiate into Tregs, at least before they were expanded in vitro culture; this decrease of potential may be the result of the allergic environment *in vivo*, but might not be affected by the cytokines in the *in vitro* environment.”

New text (lines 673-683):

“These results indicate that naïve $CD4^+$ T cells in the spleen and mLNs of Rag23-3 mice with severe allergic enteritis had a significantly reduced potential to differentiate into Tregs compared with those in mice fed the CN-diet fed the mice; this decrease of potential might be the result of changes of surface molecules expression of the cells affected by the allergic environment *in vivo* but not by the cytokines. This is consistent with the absence of detectable IL-4 or IFN-gamma in the culture supernatant of Tregs differentiated from naive $CD4^+$ T cells under polarization conditions, in contrast to the supernatant of Tregs differentiated from EMTs. Thus, this naïve $CD4^+$ T cells might be one of several lineages of naïve T cells, not a typical naïve. Therefore, we defined the naïve $CD4^+$ T cells defines as naïve-like T cells, hereafter.”
