# Supplementary material for: Ovalbumin-specific regulatory T cells differentiated from the naïve phenotype (CD44loCD62Lhi) in mesenteric lymph nodes stably suppress enteropathy even in severe food-allergic mice
Source: PLoS One. 2025 May 30;20(5):e0324105. doi: 10.1371/journal.pone.0324105 (PMC12124522; doi:10.1371/journal.pone.0324105)
Supplement: Attachment — Submitted filename: Responses to Reviewers#2.docx [file pone.0324105.s008.docx]

**Responses to the Editor’s Comments**

Comment 1

In introduction section, please explain why authors use different OVA-TCR Tg mice. At least, authors should explain what is the difference OVA23-3 and DO11.10 mice at basic levels.

Responses

As requested, we have added the following information about the differences of basic immune responses in these OVA-TCR Tg mice in the Introduction section in the revised manuscripts:

“The mice predominantly produce IFN-γ by stimulation with anti-CD3 antibody or OVA, indicating Th1-biased responses [22].” (lines 103 – 105 in the revised manuscript)

“and strong IL-4 responses, when they were stimulated with anti-CD3 antibody or OVA [22].” (lines 107 – 108 in the revised manuscript).

In addition, to clarify our aim and the differences of the 2 strains of mice, we corrected some words in the Abstract section and highlighted by yellow.

Comment 2

In Results section, authors should delete the supplementary explanation about fundamental knowledge, some sentence should move discussion section.

Responses

As the Editor commented, it might be better to move some sentences to the Discussion section.

However, we felt that it is difficult to understand our results, especially Figure 2 and 3, without the supplementary explanation of the results and the subsequent discussion of the results, so we have added the explanation at the site after the description of the results of the figures, especially Figure 2 and 3. However, in the Results section of Figure 2, we agree that the description explaining the results was not appropriate. Therefore, we have corrected some sentences (lines 434 – 435, 441– 445 as highlighted by yellow.

Therefore, we would like to submit our revised manuscript without any changes regarding the supplementary explanation, although we can understand the editor's comment.

Comment 3

it was very difficult to understand what is the significantly difference. Please use * or #, instead of a, b, c, and d, and use lines between graphs. And please change small to large alphabet, Fig1a -> Fig1A. And please change IFN-gamma -> IFNγ, TGF beta1 - TGFβ1.

Responses

We have corrected the way to show the significances by different letters, such as a, b, c, and d to * or # in all figures and each legend (highlighted by yellow) and corrected small to large alphabet like from Fig1a to Fig 1A in the revised manuscript. In addition, we have corrected IFN-gamma to IFNγ, and TGF beta1 - TGFβ1.

Comment 4

In result section, authors investigated some cytokine from helper T cells, but not IL-10 and IL-17. Why authors did not investigate IL-10 expression?

Responses

As the editor suggested, we further analyzed IL-10 production in the supernatant of MLN or spleen CD4^+^ T cells incubated under *in vitro* Treg-polarization culture condition stimulated with anti-CD3 and anti-CD28 antibodies. The results were added in Fig. 2B. The results showed that Treg-population induced in Rag23-3 or RagD10 mice differed in IL-10 production when stimulated and differentiated with anti-CD3 and CD28 antibodies.

Therefore, we have corrected the legend of Figure 2 (line 491), have added the ELISA kit used in this assay in the Methods section (lines 243 – 244) and further added the following sentences in the Results section of our revised manuscript:

“The level of IL-10 production in the spleen cells and in the mLN cells was significantly higher in the Rag23-3 mice than in the RagD10 mice when fed the EW-diet. The amount of IL-10 produced by CD4^+^ T cells in mLN was much more than that in spleen EW-fed mice.” (lines 465 – 468 in the revised manuscript)

“the production of IL-4, IFN-γ, and IL-10 in EW-fed Rag23-3 mice. In addition, Tregs differentiated from CD4^+^ T cells by stimulation with mAbs in both strains of EW-fed mice probably have sufficient regulatory function producing IL-10, but suppressive function of the Tregs in both strains of CN-fed mice may be independent of IL-10.” (lines 470 – 473 in the revised manuscript)

**Others**

We apologize for the lack of description of the result describing IL-2 production in the supernatant in Fig 5D of our previous manuscript. Therefore, we have added the following sentences in the Results section (lines 685 – 688) of the revised manuscript as follows;

“IL-2 production in mLN was comparable among experimental groups, but that in EW_EMT in SPL showed the highest level compared to those in other groups, but the difference of IL-2 production was not affected to the induction of Tregs (Fig 5D).”

**Responses to Reviewer 3**

Comment1

I suggest a revised title for the manuscript: "Stable and suppressive regulatory T cells from mesenteric lymph nodes suppress food-allergic enteropathy in mice" as the current title is long and somewhat complex.

Responses

Following the suggestion of Reviewer3, we have changed the title of our revised manuscript.

Old title: "Ovalbumin-specific regulatory T cells with the naïve phenotype (CD62LloCD44hi) from mesenteric lymph nodes suppress food-allergic enteropathy in mice, when expanded by anti-CD3/CD28 antibodies but not by ovalbumin plus antigen-presenting cells".

We agree with the critical comments of Reviewer3 that our manuscript should reduce the generality but emphasize the specificity of the method inducing regulatory T cells from the mouse model of severe intestinal allergy. However, the unique way in which we obtained the suppressive and stable regulatory T cells in this study even under severe food allergic conditions, is that by using anti-CD3 and anti-CD28 antibodies for stimulation, naive antigen-specific CD4^+^ T cells which are likely to originate from the intestinal tissues, are differentiated into Tregs. Therefore, if Reviewer3 agrees with our proposal, we think it would be better to add the words "ovalbumin-specific regulatory T cells differentiated from naïve phenotype (CD44^lo^CD62L^hi^)”, “mesenteric lymph nodes”, and “severe food allergy” in the title. Furthermore, we would like to clearly state our claim in the title, as the title suggested by Reviewer3 is similar to our short title “Stable and suppressive regulatory T cell induction in a food-allergic enteropathy mouse model”.

We would appreciate it if Reviewer3 and the Editor could reconsider and accept the title we changed based on Reviewer 3's comments in the revised manuscript as follows:

New title: “Ovalbumin-specific regulatory T cells differentiated from the naïve phenotype (CD44^lo^CD62L^hi^) in mesenteric lymph nodes stably suppress enteropathy even in severe food-allergic mice "

Comment2

Extracting Tregs from mesenteric lymph nodes (MLN) for therapeutic use in food allergy patients seems impractical. Would it not be possible to use Tregs derived from peripheral blood instead as an experimental setting which strength the requirement of their study? The manuscript appears to lack appropriate control experiments to address this question. The authors mention in line 79 that “it is difficult to separate allergen-specific Tregs from the total population of peripheral blood mononuclear cells.” However, obtaining Tregs from MLN seems even more challenging than isolation from peripheral blood mononuclear cells (PBMCs).

Responses

As Reviewer3 suggested, we agree that extracting Tregs from MLN for therapeutic use would be impossible in food allergic patients. Therefore, we proposed the use of marker molecule (such as alpha4beta7) migrating to the intestine, expressed on naïve phenotype T cells to enable the method possible in clinical use; as described in lines 828 – 830 in the discussion section of our previous manuscript;

“Therefore, our results suggest that naïve-like (CD44^lo^CD62^hi^) CD4^+^ T cells expressing a surface marker to migrate to the intestinal tissues (e.g., the gut homing integrin, alpha4beta7 [38]) may be best for producing stable Tregs”.

By considering Reviewer3’s comment, we thought that the description (line 78 – 79) in the Introduction section of the previous manuscript may give a misunderstanding to the readers of PLOS ONE, because allergic researchers have made efforts to find the maker of Tregs in peripheral blood. In addition, in the Discussion section, we mentioned about the marker migrating to the intestinal tissue of Tregs in the peripheral blood. Actually, although it was in graft-versus-host disease, C-C motif chemokine receptor 9 (CCR9), specific homing receptors for colon or small intestine, was presented as a candidate of transplantation Treg marker migrating to the intestine and reducing the symptom (American J Transplantation, 2023:23;1102-1115, <https://doi.org/10.1016/j.ajt.2023.01.030>), although they were obtained from spleen. However, they are trying to transplant Tregs migrating to the intestine and cure the disease. Therefore, we have corrected the sentence in the Introduction section and added CCR9 molecule as a candidate molecule and this paper as a reference in the Discussion section of the revised manuscript.

<Introduction section>

Old

*lines 78 – 81: however, it is difficult to separate allergen-specific Tregs from the total population of peripheral blood mononuclear cells, because of the lack of specific surface markers of Tregs with stable inhibitory functions that will not produce off-target effects, although CD137 has been proposed as a candidate marker [16-18].

New

*lines 78 – 80 in the revised manuscript: “many allergists are trying to find markers for stable Tregs specifically activated by antigens in peripheral blood, but the goal has not been achieved, although CD137 has been proposed as a candidate marker [16-18]”.

<Discussion section>

Old

*lines 828 – 830: Thus, our results suggest that naïve-like (CD44^lo^CD62^hi^) CD4^+^ T cells expressing a surface marker to migrate to the intestinal tissues (e.g., the gut homing integrin, alpha4beta7 [38]) may be best for producing stable Tregs.

New

*lines 844 – 847 in the revised manuscript: “Thus, our results suggest that naïve-like (CD44^lo^CD62^hi^) CD4^+^ T cells expressing a surface marker to migrate to the intestinal tissues (e.g., the gut homing integrin, α4β7 [38] or C-C motif [chemokine receptor](https://www.sciencedirect.com/topics/biochemistry-genetics-and-molecular-biology/chemokine-receptor) 9 specific [homing receptors](https://www.sciencedirect.com/topics/medicine-and-dentistry/homing-receptor) for colon or [small intestine](https://www.sciencedirect.com/topics/medicine-and-dentistry/small-intestine), CCR9 [39]) may be better for producing stable Tregs.”

*Reference:

**39.**	Larson JH, Jin S, Loschi M, Bolivar Wagers S, Thangavelu G, Zaiken MC, et al. Enforced gut homing of murine regulatory T cells reduces early graft-versus-host disease severity. Am J Transplant. 2023;23:1102-1115. https:// doi: 10.1016/j.ajt.2023.01.030

Comment3

The authors highlight that iTregs derived from naïve CD4+ T cells of OVA-transgenic mice strongly suppress allergies. However, their explanation about how antigen-specific naïve T cell-derived Tregs could be obtained in non-transgenic models is overly complex for a general understanding of immunological mechanisms.

There are several established mouse models for food allergy, such as OVA-Alum. It would be more robust to test the expansion of Tregs using these models, comparing the two methods described in the manuscript. Demonstrating the inhibition of allergic symptoms with the authors’ method would strengthen the study’s findings.

Responses

We are grateful for Reviewer3’s suggestion to make our results more robust as a better way to induce functional Tregs. However, we have confirmed that the food-allergic inflammation and recovery from the severe inflammation observed in the EW-fed TCR-transgenic mouse model of OVA23-3, such as enteropathy, bone loss, tolerance acquisition, and mast cell infiltration into the intestinal tissues are reproduced by a food-allergic enteropathy model established by using BALB/c mice fed with EW-diet after intraperitoneal sensitization with OVA and alum. (Burggraf M., Nakajima-Adachi H., et al. Mol. Nutr. Food Res. 2011;55:1475–1483). In our manuscript, we have not yet analyzed in detail the characteristics of Tregs in the BALB/c mouse model and therefore, we agree to confirm the reproducibility of the induction of functional Tregs in the BALB/c mouse model. Indeed, we can analyze OVA-specific Treg responses by using the BALB/c mouse model (Morinaga H., Allergol Int. 2020;69:622-625). However, even if the method of inducing Tregs used in the present study worked in the BALB/c mouse model, we would raise the question whether it was the effect of alum immunity and whether the results might be different if we used the other adjuvant. Also, in allergic diseases, effective treatments vary from person to person and may not work for everyone. Therefore, we have added a sentence in the Discussion section suggesting this Treg induction method as one of them in severe allergy and added a reference in our revised manuscript as follows;

“although we need to analyze the effect of stability of Treg function using the BALB/c mouse model [50] and verify the generality of this method,” (lines 920 – 921 in the revised manuscript)

*Reference:

**50.**	Burggraf M, Nakajima-Adachi H, Hachimura S, Ilchmann A, Pemberton AD, Kiyono H., et al. Mol. Nutr. Food Res. 2011;55:1475-1483. doi 10.1002/mnfr.201000634)

Comment4

Additionally, the manuscript cites a new reference (Reference 24). However, the cited paper’s results seem contradictory to the authors’ findings, stating that “iTregs generated by activation with antigen/APC are more suppressive than iTregs generated by anti-CD3/CD28 antibodies.” The cited manuscript attributes these functional differences to chemokine-related gene expression. If the authors choose to reference this paper, they should address this apparent discrepancy in their discussion.

Responses

Thank you for your suggestion. Although the study in reference 24 showed that the iTregs generated by anti-CD3 and anti-CD28 antibodies were less suppressive than iTregs generated by stimulation with HEL, which was the antigen of the Tregs, and APC, the situation and aim of the experiments were different from ours. We indicated in our previous manuscript that we could induce iTregs from severe allergic mice by using anti-CD3 and anti-CD28 antibodies, not antigen/APCs by the following mechanism that the mice were unable to induce Tregs when they were stimulated with antigen and APC, because of overproduction of IL-4 and IFN-γ. Therefore, we don’t think there is a discrepancy with reference24, but to avoid any misunderstanding of our aim, we have added some words in line 790 and corrected sentences in lines 794 – 796 in the revised manuscript, and both corrections are highlighted in yellow as follows;

“Although we did not investigate in detail why the responses to the Ab-stimulation were different from those to the antigen stimulation, similar findings, albeit with a different capacity of Tregs, have been reported elsewhere: Zhao et al. reported that the inhibitory effects on Th1 responses, surface molecules, and of Foxp3 expression levels in hen-egg-lysozyme–specific Tregs induced by mAbs are not the same as those induced by a combination of antigen plus antigen-presenting cells [24]. The implication of this result is that when considering adoptive Treg transfer as a therapeutic option, we need to choose the most appropriate approach to obtain Tregs with sufficient suppressive function. Our present results suggest that”

Comment 5

Overall, the manuscript leaves its conclusions unclear. It does not effectively explain what was discovered regarding the differences between the two OVA-TCR mouse strains or the mechanisms of Treg induction by different antigen stimulation methods. While the stated aim is to evaluate the stability of Tregs, the study lacks critical analysis, such as how epigenetic regulation might contribute to stabilization in the two strains of iTregs. To provide generalizable insights, I recommend additional experimental comparisons of Tregs derived from these strains to clarify their cellular properties.

Responses

We are grateful for suggesting the importance comparing epigenetic status between Tregs from Rag23-3 as a severe food-allergic enteropathy mouse model and those from RagD10 as a tolerance acquisition mouse model. However, our study got started at the finding that in Rag23-3 mice, iTregs could not be induced by stimulation with OVA plus APCs. Therefore, it is the most important finding in our study that we could obtain iTregs by stimulation with anti-CD3 and CD28 antibodies. The stability and suppressive activities of the iTreg were proved by transfer of the iTregs into EW-fed Rag23-3 mice, suggesting the iTregs would be epigenetically stable. In addition, we clarified the causes of less abilities of effective iTreg induction in our study. We did not perform the further experiment analyzing the epigenetic differences between iTregs derived from Rag23-3 and RagD10 mice, as required by Reviwer3. This is because the comparison of epigenetic state between two types of iTregs would contribute to improve the quality of the iTregs for transplantation as well as address the underlying issues related to the difference in epigenetic regulation in these iTregs, but we believe that the analysis of iTregs quality would be the next step of our study. Therefore, although we have not achieved to our ultimate goal in this study, we hope that Reviewer3 could allow our revised manuscript to be accepted without further epigenetic studies as the other Reviewers.

However, as indicated in our response to the Editor’s Comment4, in order to further explain and add to what was found regarding the differences of iTregs at the molecular level between the two OVA-TCR mouse strains, we analyzed IL-10 production in the supernatant of Tregs stimulated with anti-CD3 and CD28 antibodies and differentiated in the Treg polarization culture condition in which these T cells were derived from mLN and spleen of EW or CN-fed Rag23-3 or RagD10 mice. As a result, we could show the differences of IL-10 production among Tregs, one of the most major characteristics as Treg function. We have added the description of Figure 2B in the Results section (lines 465 – 468), and discussed the result in the Result section (lines 470 – 473) of the revised manuscript. Please see our response to the Editor’s Comment4.
